# Principles of infection prevention and reprocessing in ENT endoscopy

**DOI:** 10.3205/cto000125

**Published:** 2015-12-22

**Authors:** Axel Kramer, Wolfgang Kohnen, Susanne Israel, Sylvia Ryll, Nils-Olaf Hübner, Horst Luckhaupt, Werner Hosemann

**Affiliations:** 1 Institute for Hygiene and Environmental Medicine, University Medicine of Greifswald, Germany; 2 Hospital Hygiene of the University of Mainz, Germany; 3 Institute for Medical Diagnostics, Greifswald, Germany; 4 Dept. of Otolaryngology, St. Johannes Hospital Dortmund, Germany; 5 Dept. of Otolaryngology, University of Greifswald, Germany

**Keywords:** infection risks, reprocessing, rigid endoscopes, flexible endoscopes, spatial requirements

## Abstract

This article gives an overview on the principles of reprocessing of rigid and flexible endoscopes used in ENT units including structural and spatial requirements based on general and ENT-specific risks of infection associated with diagnostic and therapeutic endoscopy. The underlying legal principles as well as recommendations from scientific societies will be exemplified in order to give a practical guidance to the otorhinolaryngologist.

Preliminary results of a small nation-wide survey on infection control standards based on data of 29 ENT practices in Germany reveal current deficits of varying degree concerning infection control management including reprocessing of endoscopes. The presented review aims to give support to the establishment of a structured infection control management program including the evaluation of results by means of a prospective surveillance.

## 1 Introduction

During the last decades, endoscopic procedures have opened completely new perspectives for diagnostics and therapy in otolaryngology by direct access to the examination fields. Among others, one precondition of the safe use of ENT endoscopy is the observation of hygienic standards of reprocessing of the instruments and the compliance with general principles of infection prevention during endoscopy.

The following explanations are based on a series of basic recommendations:

1. Joint recommendation of the Commission for Hospital Hygiene and Infection Prevention at the Robert Koch Institute (KRINKO) and the Federal Institute for Drugs and Medical Devices (BfArM) on the hygienic requirements regarding the reprocessing of medical devices [1].
2. Joint guideline of the German Society for Hospital Hygiene (DGKH), the German Society for Sterile Supply (DGSV), the Working Group Instrument Reprocessing (AKI), and the Association for Applied Hygiene (VAH) on the validation of manual cleaning and manual chemical disinfection of medical devices [2].

With special consideration of those recommendations, the particular preconditions for the hygienically safe use are explained and the requirements of a secure reprocessing of ENT specific endoscopes are described with hints to special risks and future challenges.

## 2 Infection risk by diagnostic and therapeutic endoscopy

Repeated diagnostic and therapeutic endoscopies may bring in pathogens of nosocomial infections by the use of the endoscope if no reliable reprocessing is performed after its use. Furthermore, regularly reprocessed endoscopes might be contaminated by the staff, the working environment, or the patients and thus represent a source of contamination if the basic principles of infection prevention are not observed.

It depends on the type and extent of tissue traumatization, the local and systemic defense of the patient, and the pathogen, if infection develops after the application of a contaminated endoscope. If the protective mucosal barrier is injured during an endoscopically assisted intervention, the pathogens can enter from exterior or from the surface bacteria, possibly even via the endoscope into the tissue. Thus the infection risk increases with the invasiveness of the intervention [3]. However, even during rhino-neurosurgical surgeries with their inevitably broad opening of the dura, the associated risk is relatively low: the rate of intracranial infections in those maximized interventions is given with 1.6% and the infection-related mortality with 0.125%. It is comparable to the one of transcranial surgeries without endoscope [4].

Nosocomial pathogens in the ENT field found in the nasal vestibulum are mainly *Staphylococcus aureus* and MRSA but also *Streptococcus pneumonia* and other *Streptococcus* spp., *Candida albicans*, and Gram-negative bacteria such as *Klebsiella* spp., *E. coli, Proteus* spp., *H. influenza*, and *P. aeruginosa* as well as representatives of the resident flora such as *Bacteriodes* spp. and *Corynebacterium* spp. [5]. In 2003, the Creutzfeld-Jakob prion protein (PrP^Sc^) was found for the first time in cadavers with sporadic CJD in the olfactory epithelium [6], so that the KRINKO-BfArM recommendation [1] defined the olfactory epithelium as high risk tissue in ENT surgery. Examinations of nasal brushing specimens indicate that further evaluations of this situation are required [7].

Different microbial habitats of more than 25,000 species are found in the oral cavity [8], [9]. In the free oral cavity, aerobe organisms are predominant while for example in the subgingival space anaerobes such as *Actinomyces, Fusobacteria, *and* Peptostreptococci* are found. Viruses in the upper respiratory tract that are particularly relevant are rhinoviruses, coronaviruses, Coxsacki A viruses, influenza A viruses, respiratory syncytial viruses, adenoviruses, and para-influenza viruses, but also enteroviruses [10], [11]. 

The paranasal sinuses and the larynx are physiologically not colonized despite the density and multitude of microorganisms found in neighboring biotopes [12].

In contrast to gastroscopy, bronchoscopy, and arthroscopy [13] the data situation regarding the infectious risk of ENT specific instruments is rather scarce [14], the irregular reprocessing of instruments is supposed to cause a high infection risk. This thesis is supported by the results of an investigation of laryngoscopes used in anesthesia that were simply wiped with disinfectant tissues between the applications [15]. Under those conditions, the instruments were contaminated after insufficient reprocessing in 22% and before use (because of meanwhile occurred contamination) in 86% of the cases with critical pathogens [16].

## 3 Principles of infection prevention for diagnostic and therapeutic endoscopy

Each endoscopically assisted surgery requires careful indication and planning of the intervention also with regard to hygienic aspects. Patients who have a high risk of MRSA or MRGN colonization should be identified and examined microbiologically short-term before elective interventions if the mentioned pathogens bear the risk of additional danger for the patient [17], [18]. Visceral surgery has already implemented this concept [19]. The ambulant screening of MRSA (not of MRGN) is already specially paid if applied in high risk patients since April 2012 in Germany. High risk patients in this context are people who have been hospitalized during the past 6 months for at least four consecutive days and who meet additionally the following criteria: known MRSA history and/or at least one of the following risk factors: chronic need for care (at least nursing category 1), antibiotic therapy within the past six months, catheterization (e.g. urinal catheter, PEG tube), patients requiring dialysis, skin ulcus/gangrene/chronic wounds/deep soft tissue infections. Those statements do not apply to diagnostic endoscopy.

Regarding diagnostic endoscopy, the following aspects must be considered. Via patient contact surfaces, working surfaces, and surfaces of medical devices pathogens may reach a patient by using hands or also by direct patient contact [20]. So the disinfection of the hands and surfaces near the patient are essential as part of basic hygiene.

Before purchase of each medical device it is necessary to think about the performance and the efforts and costs of the reprocessing. The user has to write down for every medical device if, how often, and with which procedure it will be reprocessed.

According to the German law ([21], §4), generally the reprocessing of medical devices that are properly applied with low microbiological contamination or sterile has to be performed with consideration of the manufacturer’s information with appropriate validated procedures so that the success of those procedures is traceably assured; and the security and health of patients, users, or third parties is not endangered. An appropriate reprocessing is assumed when the KRINKO-BfArM recommendations [1] are observed. The requirements have to be applied to the outpatient and the inpatient sectors.

In accordance to the use and the material properties, endoscopes must be reprocessed before they are used again. The suitability and effectiveness of the reprocessing procedures must be clarified by product-specific validated tests [1]. The validation proves that with observation of the determined process parameters and methods under different conditions a defined quality of reprocessing is always traceably achieved.

For determination of the reprocessing, endoscopes have to be classified according to the specific requirements of reprocessing. The user is responsible for the correct classification of the medical devices in consideration of the manufacturer’s data. Endoscopes used in otorhinolaryngology do not have a working channel, they are only in contact with (probably pathological) mucosa or skin and as they do not penetrate them during diagnostic measures, they are classified as medical device of the category “semicritical A”. For the reprocessing of medical devices classified higher than “semicritical A”, the medical and nursing staff have to dispose of a special knowledge that can be acquired in the context of special trainings and courses, e.g. certificate of competence in “processing of endoscopes” or other courses with a curriculum in accordance to DGSV.

Based on the KRINKO-BfArM recommendation [1], automatic as well as manual reprocessing are generally allowed for ENT endoscopes with diagnostic use. Still, automatic reprocessing is preferred due to the reduced time and personnel costs and an easier standardization. While the validation of automatic reprocessing is often performed by the manufacturer [22], [23], validation of manual reprocessing (including every single step) must be provided by the user himself or on his behalf. Disinfectants must be VAH-certified and recommended by the manufacturer to ensure effectiveness as well as compatibility with the materials of the medical device. The same principle is applied to previous cleaning and final rinsing, i.e. every single step of the whole process must be defined, validated, and determined in an SOP. Considering the manufacturer’s data, the responsible person writes down and determines the procedures that are applied (every single step) and the conditions (e.g. rooms, equipment, qualification of the staff) under which his medical devices are reprocessed and stored. The SOPs have to mention explicitly all critical steps of the process. They are periodically checked in order to prove the continuous effectiveness.

The guideline for validation of manual cleaning and manual chemical disinfection of medical devices describes in detail the specific method [2]. For establishing of an SOP, the following conditions must be observed:

- The procedure must be specified sufficiently. The specification includes a detailed description of all consecutive steps such as pre-treatment, pre-cleaning, cleaning, intermediate rinsing if required, disinfection, final rinsing, drying, check of cleanness/intactness, and functional test.
- The effectiveness of every single reprocessing step has to be documented in consideration of the “worst case” situations in the context of validation.

Cleaning as first step of reprocessing has to be performed imperatively with non-fixing agents because otherwise coagulated protein could stick to the medical device, a situation that might question the effectiveness of the subsequent disinfection [24]. That is why the application of glutaral and peracetic acid are not recommended for pre-cleaning and cleaning [25]. According to the KRINKO-BfArM recommendation [1] the disinfectant has to have a bactericidal (including mycobacteria), fungicidal, and virucidal effect. In contrast, the guideline of the DGKH, DGSV, AKI, and VAH regarding the manual reprocessing contains reduced requirements concerning the range of effects [2]. If the olfactory epithelium is penetrated or biopsies are taken from the olfactory region, the view is taken that reprocessing should also assure the effectiveness against prions [1]. With regard to the still unknown actual risk of this aspect, these circumstances should be analyzed more intensively in order to avoid writing down possibly exaggerated and precipitate demands. 

For start-up and operation of cleaning and disinfection devices the check lists of the KRINKO-BfArM recommendation [1] with their enclosures 3 and 4 are helpful. The requirements for the device, the process validation (consisting of installation, operation, and performance qualifications), the periodical or batch-related routine checks, the measurement supervision and the control of the process parameters, the periodical checks of the procedures (re-evaluation of the performance), and the event-related checks of the procedures (performance assessment based on a specific event) are listed there. Regarding the performance qualification, evidence has to be provided that after manual cleaning and disinfection reproducibly cleaned and disinfected medical devices result in accordance to the operating procedures.

## 4 Manual reprocessing of rigid and flexible endoscopes

In the following, an example will be given for the hygienic requirements of sufficient manual reprocessing of rigid and flexible endoscopes. The validation of this procedure must always be implemented on the own responsibility, alternatively there are external validators at disposition.

The manual reprocessing is performed in four steps with consideration of staff protection measures and other product information provided by the manufacturer.

- Manual pre-cleaning/cleaning immediately after examination considering the staff protection (gloves, protective gowns, and eye protection): directly after removal of the endoscope at the end of the examination, the inserted part is wiped for example with water-moist tissue in order to remove larger impurities and to avoid the surface drying of organic material. After that, the endoscope is rinsed with tap water and dried with lint-free single-use wipes. This procedure is meant to avoid diluting of the disinfectant in the second step. If the olfactory epithelium was definitely penetrated during endoscopy, the endoscope should be put into prion effective agent after pre-cleaning as intermediate step, for example 6 mol GdSCN solution for 15 minutes [26]
- Afterwards disinfection is performed: the cleaned endoscope is put into the disinfectant solution (options for virucidal agents and application time according to manufacturer’s data). Appropriate trays have to be used. The instruments have to be fully covered by the solution (except the ocular with nozzle for light). No hollow spaces or air bubbles must occur. The predetermined application times and concentrations have to be observed. The handle of the endoscope is cleaned with the same disinfectant that is used for the instrument. The disinfection solution is changed every day and additionally in cases of visible contamination. The trays have to be cleaned and disinfected every day, too, as well as in cases of visible contamination.
- The third step consists of the final rinsing: after disinfection, the endoscope is rinsed carefully with tap water. As the quality of the water cannot be assured unless continuous microbiological surveillance is performed, terminal sterile filters are necessary [1]. Rinsing is meant to remove remaining disinfectant. The recommended filters can be left in place for 3 months [27].
- Finally, the external part of the endoscope is dried with a fresh single use wipe and visually checked for clean and intact appearance.
- Up to the next use, the endoscope has to be stored in a dry and dust-protected place.

It is clearly not admitted to wipe medical devices (laryngocopes) only with alcohol-soaked compresses after use.

For automatic reprocessing, the endoscope is put into the cleaning and disinfecting device after manual pre-cleaning.

## 5 Spatial requirements of ENT specific examination rooms

Forward-looking planning of the spatial environment contributes to the effectiveness of hygienic measures. Compared to hygiene habits and the appropriate reprocessing of endoscopes, this contribution to infection prevention is rather low unless the reprocessing process is not impaired by spatial circumstances.

It must be possible to clean wet and disinfect all surfaces in the examination room. The examination rooms must offer sufficiently free moving in order to avoid cross contamination between the dirty and clean zone in the area of reprocessing or between patients and their surroundings.

If a sufficiently big working surface is available for reprocessing endoscopes that allows separation into an dirty and clean area [1], the reprocessing can be performed in the examination room. For manual reprocessing, a double sink is recommended in order to avoid airborne recontamination during final rinsing (performed on the “pure” side) [28].

## 6 Preliminary results of a survey based on a questionnaire regarding the reprocessing of endoscopes in ENT institutions

From 2013 to 2014, 696 questionnaires were sent out to ENT institutions for collecting data on the hygiene status. The answers of 15 private practices, 8 community practices, and 6 institutions without data on the structure of the practice from all over Germany could be evaluated. In 9 institutions, outpatient surgeries, and in 4 institutions inpatient surgeries were performed. The return rate of 3% is noticeably low; it justifies only a first orienting evaluation.

The questions were related to the organization of the hygiene in the practices, spatial preconditions, staff protection, hand hygiene, assessment of infection anamnesis, perioperative antibiotic prophylaxis, use of antiseptics, presence of antibiotic guidelines, surveillance of SSI, reprocessing of medical devices with special consideration of ENT specific treatment units as well as reprocessing of rigid and flexible endoscopes, sterilization procedures, storage of sterile goods and surveillance and documentation of hygienic measures including water safety. The following description focuses on the data of the questionnaire revealing the reprocessing of medical devices. Information on hand disinfection is added because deficiencies in hand hygiene might be an origin of contamination of already reprocessed endoscopes.

Regarding hand hygiene, partly severe deficiencies could be identified (see Table 1 (Tab. 1)). The table reveals that hand disinfection is considered more important for self-protection because the hands are disinfected always after treatment of high risk patients and after contamination, but not before and after every treatment of patients. It is critical that some staff members have artificial or gelled fingernails. There are several reasons to refuse this habit. The density of bacteria on those artificial nails is much higher than on natural ones. At the same time, artificial nails mislead to neglecting washing of the hands and they increase the risk of perforating the protective gloves [29], [30], [31], [32], [33]. Repeatedly, artificial nails could be identified as reason for surgical site infections [34], [35], [36]. Jewelry for hands and forearms hinder proper hand hygiene and may be a reservoir for pathogens. A correlation between the number of rings and the number of Gram-negative bacteria and *Staph. aureus *could be found in the intensive care staff [37]. When the nurses had rings on their fingers, an increased rate of enterobacteriaceae and non-fermenters could be found on the hands, however, an increased transmission rate could not be revealed [38], [39]. Finally, rings increase the risk of injuries as well as the rate of perforations for single-use gloves [40], [41].

Putting those data in relation to the conditions of reprocessing of medical devices, several deficiencies become obvious. They are for example due to a lack of competence and expertise (see Table 2 (Tab. 2)). Other specific articles identify the missing instructions and SOPs as severe deficits [42]. Such deficits may lead for example at the occasion of inspections by health authorities to important measures such as the outsourcing of reprocessing until appropriate instructions are elaborated.

Regarding the reprocessing of rigid and flexible endoscopes, the survey reveals – despite the aspect of the few responses – that the hygienic requirements of reprocessing endoscopes are not observed to an adequate extent (see Table 3 (Tab. 3)). The endoscopes are mostly reprocessed manually. This fact corresponds to data in the literatures: a survey of private practices performing outpatient surgeries (including ENT practices) made by the health authorities of Frankfurt/Main, Germany, showed that the reprocessing of instruments was carried out manually in 93% [43]. In dental practices the situation in 2005 with manual reprocessing was slightly better [44]. A report published 2008 by the Federal Ministry of Health also drew the conclusion that cleaning and disinfection in private practices is nearly always performed manually. The reason is the financial burden [45]. The statement is given that changes of these suboptimal procedures would require specific official instructions [43]. In this sense, the new infection protection law of 2011 [46] and the updated hygiene regulations increase the requirements for the structural and process quality of hospital hygiene. The users have to face stricter planning and documentation obligations with tighter reporting duties, a supervision by the public health service was established [47].

## 7 Tasks in the reprocessing of ENT specific endoscopes

Endoscopy did not only enlarge the treatment options in otorhinolaryngology but it also made efficient minimally invasive procedures possible. In order to fully understand the benefit of this technique it is necessary to know about the risks associated with thin intervention and to avoid undesired events. Infections are an important part of those undesired events.

The current hygiene standards seem to allow the reduction of the rate of known infections to a minimum. Thus a lowering of the standard must not be discussed. In contrast, increasing claims of patient safety always have to be in a balanced relationship to the affordability of the medical services. Hygienic aspects have to be integrated effectively and economically in the treatment procedures. Especially functional units with a high rate of interventions and patient flow, optimized processes implementing hygiene and patient safety play a decisive role.

Generally, automatic cleaning and disinfection procedures are preferred because of the higher safety [48] and reproducibility and because of the staff protection. The manual reprocessing of endoscopes for diagnostic use, however, is allowed unless the whole process is validated. For revalidation, there are no timely requirements; generally the interval is suggested by the validator.

According to the Medical Device Directive [21], the manufacturer of medical devices is obliged to define the reprocessing procedures on the basis of the process validation. Unfortunately, there are partly enormous deficiencies regarding the definitions of manufacturers [49].

The responsibility for hygiene belongs to the physician or the director of the institution. He can delegate hygienic measures according to the qualification of the staff members.

In every institution performing surgeries a link physician for infection control must be nominated up to 2016 (depending on the federal state, it may even be earlier) [46] who has passed a basic course on hygienic matters of 40 hours or an e-learning curriculum. This does not apply to institutions where only diagnostic endoscopies are performed. The hygiene plan must contain detailed measures according to the peculiarities of the institution [46]. Further, this hygiene plan has to distributed and explained to all staff members at the occasion of their employment, which has to be documented. If changes occur in the working field or new products or processes are introduced, the hygiene plan has to be changed accordingly. The instructions of the hygiene plan have to be repeated and documented regularly – at least once per year [46].

For surgically working institutions the prospective clinical surveillance of postoperative wound infections is obligatory. To meet the requirements it is recommended to choose a representative marker operation [50], [51]. 

## 8 Conclusion

The reprocessing of medical devices is regulated by the Medical Devices Law [52] and the Medical Device Directive [21]. Further detailed requirements are defined in the KRINKO-BfArM recommendation [1] that was explained extensively in a booklet published by the Association of Statutory Health Insurance Physicians of Bavaria [53].

Regarding the quality management of reprocessing the following instructions and requirements can be deduced:

- According to § 135a of the penal code V [54], there is an obligation for quality management in a private practice, however, certification is not necessary.
- Every year an instruction has to be given to the staff members regarding the reprocessing of medical devices.

Only clear regulations of the hygiene management can assure a continuously high quality of hygiene. But it must be considered that exaggerated or unrealistic hygiene requirements may be counterproductive because of economic constraints and the necessary compliance of the people involved and thus they should be avoided.

## Abbrevations

- AKI: Working Group Instrument Reprocessing
- BfArM: Federal Institute for Drugs and Medical Devices
- CJD: Creutzfeldt Jakob disease
- COEN: Compliance and Enforcement Group
- DGSV: German Society for Sterile Supply
- DKGH: German Society for Hospital Hygiene
- GdSCN: Guanidine thiocyanate
- KRINKO: Commission for Hospital Hygiene and Infection Prevention at the Robert Koch Institute
- MP: Medical device
- MRGN: multiresistant Gram-negative bacteria
- ÖGD: Public Health Service
- RDG: washer-disinfector
- SOP: standard operating procedure
- SSI: surgical site infection
- VAH: Association for Applied Hygiene
- vCJD: variant Creutzfeldt Jakob disease

## Competing interests

The authors declare that they have no competing interests.

## Figures and Tables

**Table 1:**
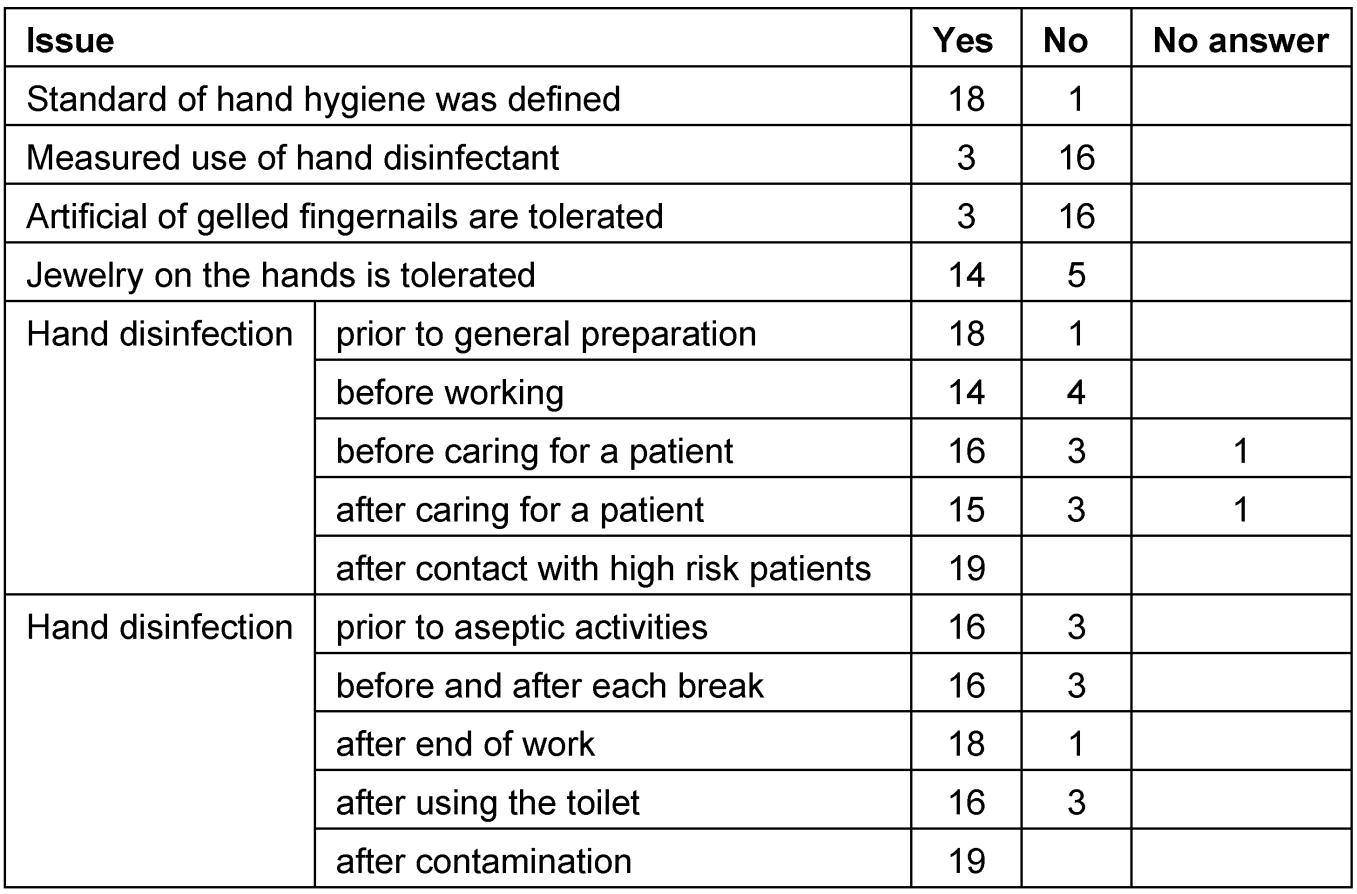
Results of a survey on hygiene standards in ENT practices: data on hand hygiene

**Table 2:**
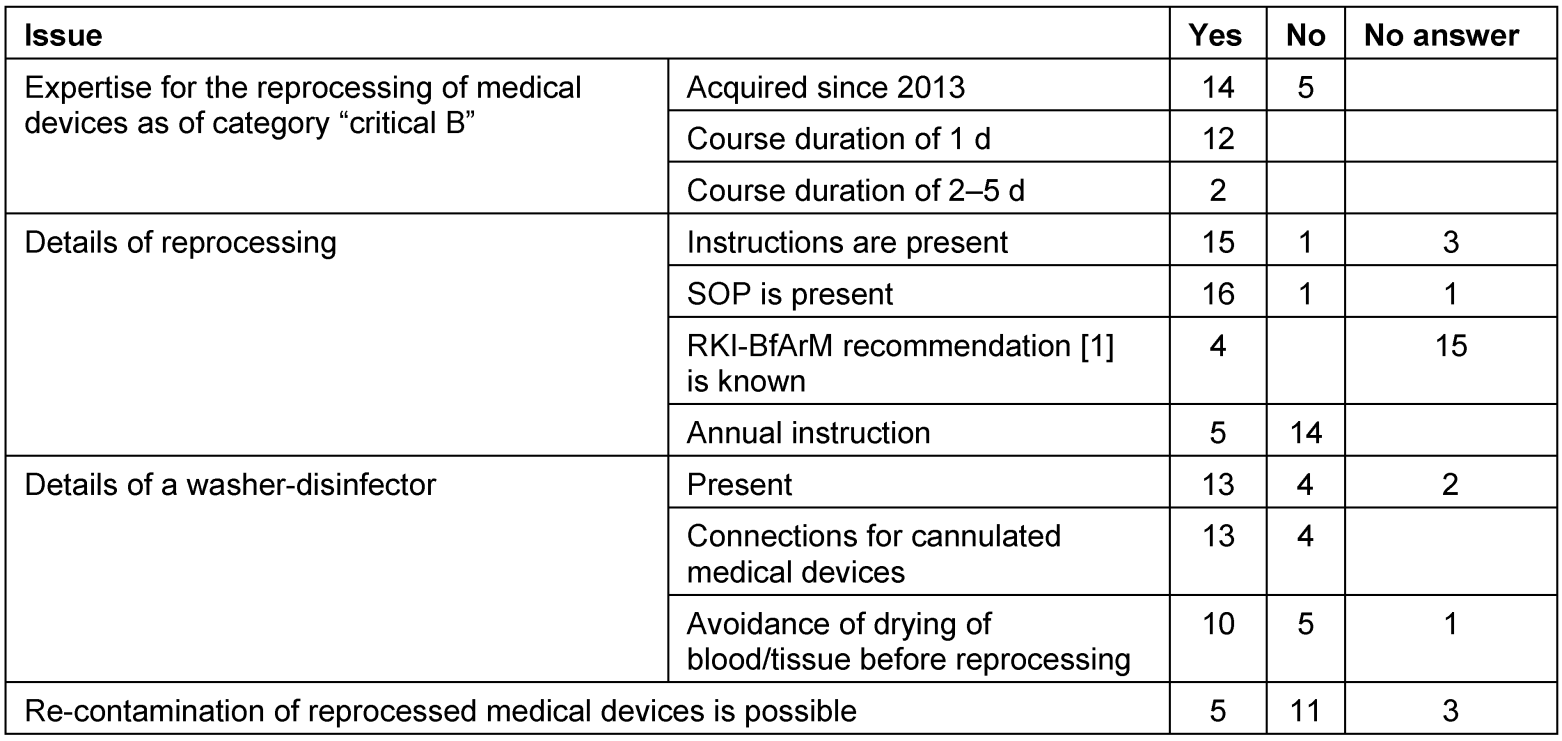
Results of the survey on hygiene standard in ENT practices: data on preconditions for reprocessing medical devices

**Table 3:**
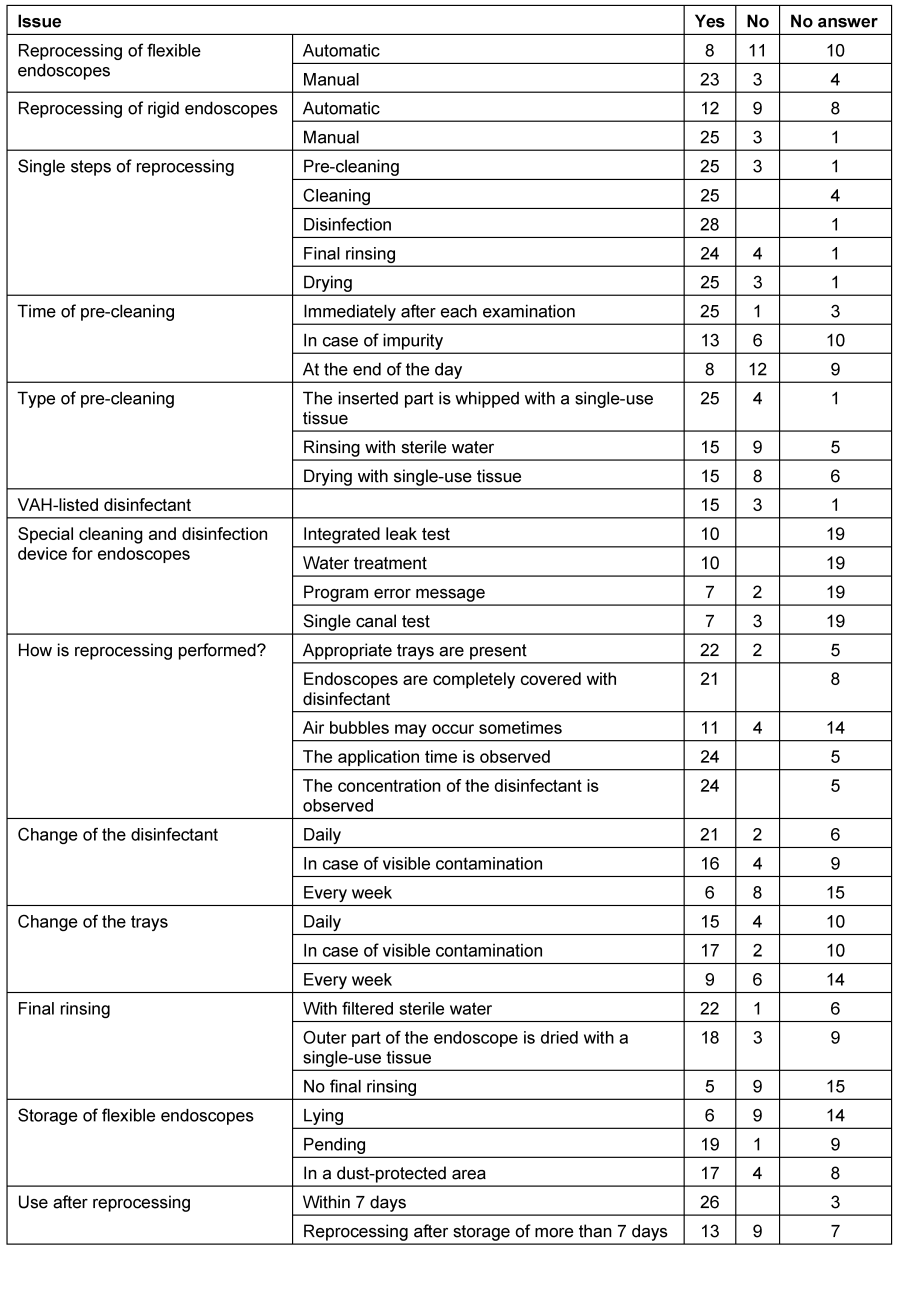
Results of the survey on hygiene standard in ENT practices: data on reprocessing rigid and flexible endoscopes
